# Bridging Gaps in Standards for the Secondary Use of Health Data: Exploratory Standard, Tool Assessment, and Feasibility Study

**DOI:** 10.2196/88619

**Published:** 2026-09-22

**Authors:** Jaleh Shoshtarian Malak, Leonie von Berlin, Irina Balaur, Rada Hussein, Philip Gribbon, Yojana Gadiya, Somayeh Abedian, Andreas Kremer, Stefan Sauermann, Francisco J Núñez-Benjumea, Jens Declerck, Sumit Madan, Hanna Ćwiek-Kupczyńska, Lukas Rohatsch, Holger Fröhlich, Christian Muehlendyck, Venkata P. Satagopam

**Affiliations:** 1 Luxembourg Centre for Systems Biology University of Luxembourg Belvaux Luxembourg; 2 Discovery Research ScreeningPort Fraunhofer Institute for Translational Medicine and Pharmacology Hamburg Germany; 3 Ludwig Boltzmann Institute for Digital Health and Prevention Salzburg Austria; 4 Bonn-Aachen International Center for Information Technology (B-IT) University of Bonn Bonn Germany; 5 Institute of Health Policy, Management and Evaluation University of Toronto Toronto, ON Canada; 6 Information Technology for Translational Medicine S.A Esch-sur-Alzette Luxembourg; 7 Faculty Life Science Engineering FH Technikum Wien Vienna Austria; 8 Institute of Biomedicine of Seville IBiS/Virgen Macarena University Hospital University of Seville Seville Spain; 9 Department of Public Health and Primary Care Unit of Medical Informatics and Statistics Ghent University Ghent Belgium; 10 The European Institute for Innovation through Health Data Ghent Belgium; 11 Fraunhofer Institute for Algorithms and Scientific Computing (SCAI) Sankt Augustin Germany; 12 Faculty Computer Science & Applied Mathematics FH Technikum Wien Vienna Austria; 13 Institute for Digital Medicine University Hospital Bonn Bonn Germany; 14 Lamarr Institute for Machine Learning and Artificial Intelligence University of Bonn Bonn Germany; 15 Johnson & Johnson Medical GmbH Norderstedt Germany

**Keywords:** EHDS, FAIR principles, health data standards, OMOP-CDM, secondary use of health data

## Abstract

**Background:**

Standards for the secondary use of health data remain under development and do not comprehensively address specific categories of health data. Existing health data and interoperability standards are primarily designed for the primary use of health care, leaving gaps in their applicability for research, regulatory and decision-making. This research is part of the Integration of Heterogeneous Data and Evidence Towards Regulatory and Health Technology Assessment Acceptance (IDERHA) project, which creates a federated processing environment for secondary health care use cases.

**Objective:**

This study aims to recommend a set of standards and tools to support harmonization objectives in the secondary use of health data. The proposed standards establish a shared representation model for data providers and stakeholders, improving data adherence to findable, accessible, interoperable, and reusable (FAIR) principles and interoperability.

**Methods:**

In this exploratory assessment and feasibility study, we performed a consensus-based evaluation of health data standards and harmonization tools across major health-data categories. State-of-the-art standards from different standard-producing organizations were evaluated based on community adoption, technical suitability, and alignment with European Health Data Space requirements. Data-harmonization tools were tested in a pilot experiment using synthetic Observational Medical Outcomes Partnership (OMOP) Common Data Model (CDM)–based data to assess the quality and feasibility of applying harmonization workflows within IDERHA. We also assessed the inherent adherence to FAIR principles of the OMOP-CDM, identifying its strengths in semantic interoperability and data reuse. Finally, we addressed the datasets handling challenges (metadata discoverability, global identifiers, access control, and licensing), which are not inherently solvable by bare data description standards, through complementary mechanisms implemented in IDERHA.

**Results:**

We recommend a set of standards to support the secondary use of heterogeneous health data across clinical, imaging, genomic, socioeconomic, environmental, and patient-reported outcome measure (PROM)/patient-reported experience measure (PREM) domains. This standards collection, centered on OMOP-CDM and complemented by established extensions, was paired with selected harmonization and quality-assessment tools. Feasibility testing on synthetic OMOP data confirmed the suitability of the tools for evaluating data quality, mapping accuracy, and extract, transform, and load. A lung-cancer use case demonstrated how diverse datasets can be harmonized and prepared for federated analysis. A FAIR-dataset maturity evaluation showed that integrating OMOP data into the IDERHA environment substantially improved adherence to FAIR principles—particularly in metadata completeness, interoperability, and hosting capabilities—supporting enhanced discoverability, accessibility, and reuse of standardized health data.

**Conclusions:**

By strengthening adherence to FAIR principles, this research facilitates the broader integration of standardized health data for regulatory processes and health technology assessment applications. The proposed standards and tools provide a comprehensive overview of existing approaches, while offering solutions that can be applied to other scenarios involving the secondary use of health data.

## Introduction

The secondary use of health data involves processing medical information for purposes beyond individual patient care, including research to support decision-making, advancing health care processes, and optimizing resource allocation. It spans 3 key domains: policy and regulatory decision-making, biomedical research, and technical innovation, including applications of AI and machine learning (ML) [1]. Many countries have developed their infrastructure and systems for managing (ie, collecting, processing, and analysis) the secondary use of health data. However, there is a need to increase the capacity of standards and guidelines to enhance data quality [2]. Currently, the European Health Data Space (EHDS) [3] is meant as the primary ecosystem managing the European strategy for health data, and all the European Union (EU) countries are working toward implementing this initiative. One of their main objectives is to enable the reuse and sharing of health data through persistent and reliable rules, practices and standards for the secondary use of data, fostering research and innovation. Several complementary initiatives have also aligned with these goals and contributed to their technical and policy-level implementation. One example is the Joint Action Towards the European Health Data Space (TEHDAS) [4] and the subsequent projects [5], which offer guidelines and technical details for promoting and accessing primary and secondary health data for boosting citizen care, health care management and research across EU countries. This project involves establishing standards for safe data processing, creating data models for collaboration, and outlining guidelines for data discovery, access and quality. These developments also highlight the increasing demand for infrastructures capable of supporting federated AI/ML, where data remain at source while enabling collective intelligence and secure model training across organizations [6]. Similarly, the Genomic Data Infrastructure (GDI) [7] seeks to enable access to genomic, phenotypic and clinical data across Europe by providing federated data access and data discovery (using the Data Catalog Vocabulary (DCAT) Application Profile for Data Portals in Europe (DCAT-AP) [8].

The Observational Health Data Sciences and Informatics (OHDSI) [9] community offers open-source solutions for the standardization of observational data to facilitate further analysis, decision-making and generating real-world evidence. In this effort, they developed the Observational Medical Outcomes Partnership (OMOP) Common Data Model (CDM) [10] to provide a unified data model and associated vocabularies for sharing and integrating health data from diverse sources to promote collaborative research. The OHDSI community has formed several work groups to extend the OMOP-CDM to cover more aspects of health-related data, such as OMOP-CDM Medical Imaging [11] and Genomic-CDM [12]. Expanding on these efforts, the European Federation for Cancer Images (EUCAIM) [13] initiative developed a pan-European digital federated infrastructure of findable, accessible, interoperable, and reusable (FAIR) [14] cancer-related, deidentified, real-world images in compliance with OMOP-CDM, along with other common standards such as Fast Healthcare Interoperability Resources (FHIR) [15], Digital Imaging and Communications in Medicine (DICOM) and DCAT-AP, to advance AI technologies for cancer diagnosis and treatment. Additionally, using OMOP-CDM for federated retrospective data analysis, there are several initiatives, including the Data Analysis and Real World Interrogation Network (DARWIN) EU [16] (established by European Medicines Agency [EMA] [17] and the European Medicines Regulatory Network [EMRN] [18]), Optimal Treatment for Patients With Solid Tumours in Europe Through Artificial Intelligence (OPTIMA) [19], EUCAIM and Health Outcomes Observatory [20] project, that aim to deliver real-world evidence from across Europe. Overall, the OMOP-CDM covers many aspects of data, which are today routinely collected in clinical practices; however, the canonical CDM does not include other types of health care related data, leading to potential gaps for some use cases [21,22]. Currently, there are some extensions created and led by various communities under the supervision of OHDSI to cover different domains of data, including imaging, geographic information system (GIS), genomic data and medical device–related data [23]. However, the OMOP-CDM extensions are still under development and not widely tested for different scenarios. Also, differences in local practices and terminology may cause inconsistencies. While OMOP offers broad coverage, mapping health data to the OMOP model is complex, especially when the source data differs in granularity and structure and does not have an exact match [24]. Currently, the European Health Data & Evidence Network (EHDEN) Academy and OHDSI community provide educational resources and certification for data models and tools related to real-world health data and evidence.

Launched in 2023, the Integration of Heterogeneous Data and Evidence Towards Regulatory and Health Technology Assessment Acceptance (IDERHA) [25] is an EHDS-aligned project designed to create an infrastructure where data holders can make their standardized datasets available for federated processing in secondary health care use cases. The project establishes a federated data access ecosystem for running disease agnostic data analysis algorithms (including federated AI/ML). IDERHA creates a secure, privacy-protected environment for collaboratively running ML algorithms across multiple clients in federated mode—which means that, instead of transferring the data, a global ML model is trained across multiple clients and updated on a central server by iteratively aggregating local models (more specifically, gradients of model parameters) from the client nodes [22,26].

Initially, IDERHA will focus on lung cancer data, and will support AI-based individualized risk profiling, malignancy detection, and (optionally) also prognosis. Given that lung cancer is one of the leading causes of cancer deaths around the world [27], it is a suitable use case for exploring federated learning in IDERHA. Alongside development of the federated ecosystem, IDERHA aims at structuring, sharing and preserving data aspects and infrastructure, ensuring that its developments and research impact extend beyond the funding period and maximizes the value of investments by preventing redundant efforts. By adopting FAIR data practices, the project fosters scientific progress, cross-disciplinary collaboration, and data reuse, ultimately enhancing reproducibility [28].

This study adopts an exploratory standard and tool assessment and feasibility approach, comprising three components: (1) consensus-based assessment of health data standards and harmonization tools across multiple domains, (2) assessment of adherence to FAIR principles of IDERHA platform, and (3) a feasibility experiment using synthetic OMOP-CDM data. Specifically, the study aims to (1) select a set of data standards across multiple categories of data (including clinical data, imaging, socioeconomic data, environmental data, patient-reported experience measures [PREMs] and patient-reported outcome measures [PROMs], molecular biology and genetics, metadata, and security and privacy) for secondary use of health data, (2) propose a set of standard-compliant data preparation and harmonization tools, (3) demonstrate a use case scenario for lung cancer disease risk assessment to show how heterogeneous data comply with the standards and how the tools are used, (4) test the feasibility of a subset of the proposed tools—focused on data quality and readiness assessment—in a pilot experiment using synthetic data provided by our data holders, and (5) assess the improvement in adherence to FAIR principles contributed by IDERHA by comparing the inherent adherence to FAIR principles of OMOP-CDM data in isolation against the same data implemented within the IDERHA platform. The results of this research could guide other research focused on the secondary use of health data, enhancing data management and improving interoperability in accordance with EHDS regulations.

## Methods

### Study Design

This research follows an exploratory standard and tool assessment and feasibility study, organized in four sections: (1) consensus-based assessment and selection of health data standards and harmonization tools across multiple data domains, (2) qualitative and quantitative assessment of adherence to FAIR principles of IDERHA platform using the FAIR-dataset maturity (DSM) framework, (3) a feasibility experiment using synthetic OMOP-CDM data to test selected harmonization tools, and (4) an illustrative use case demonstration of personalized lung cancer risk assessment.

### Ethical Considerations

Our study involves assessments of tools on synthetic clinical datasets, provided to IDERHA by our Andalusian partners—Servicio Andaluz de Salud. The Andalusian Ethics Committee is governed by Decree 8/2020 of January 30, which regulates health care ethics and biomedical research ethics in this region. The decree stipulates that clinical research must ensure the protection of participants in accordance with the ethical principles of the Declaration of Helsinki and the relevant Council of Europe Convention. Thus, the study for the generation of synthetic data was developed in accordance with these ethical principles and good research practice. The reference code assigned to the communication/application was SICEIA-2026-000464. A waiver of informed consent was requested in accordance with the Seventeenth Additional Provision, Processing of Health Data. Section d) of Organic Law 3/2018 of 5 December on the Protection of Personal Data and the Guarantee of Digital Rights.

### Standard and Tool Assessment

As shown is Figure 1, this study follows a consensus-based assessment approach to identify and select data standards and harmonization tools across the health data categories relevant to IDERHA. We first identified the main categories of health data to be used in IDERHA based on the project use cases and a characterization of data types held at participating data-holder sites (hospitals, cancer registries, research institutions, and biobanks) through structured discussions within the IDERHA data standards, technical platform, and AI modeling work packages. For each category, we then performed a structured review of candidate standards drawing on the following sources: (1) registries, technical specifications, and recommendations from established health data communities and standards development organizations, including OHDSI, ISO, World Wide Web Consortium (W3C) [29], Health Level Seven (HL7), Clinical Data Interchange Standards Consortium (CDISC), the eTRIKS StarterPack [30], and Infrastructure for Spatial Information in the European Community (INSPIRE); (2) published literature and project deliverables from related EU initiatives, including TEHDAS [5], EUCAIM, GDI, DARWIN EU, and the Pilot for a European Health Data Space on Secondary Use of Health Data; (3) structured expert input from IDERHA consortium members across 4 work packages, collected through a series of dedicated internal meetings held between 2023 and 2025; and (4) alignment assessments against the EHDS regulatory requirements and the European Interoperability Framework layers as operationalized by TEHDAS.

**Figure 1:**
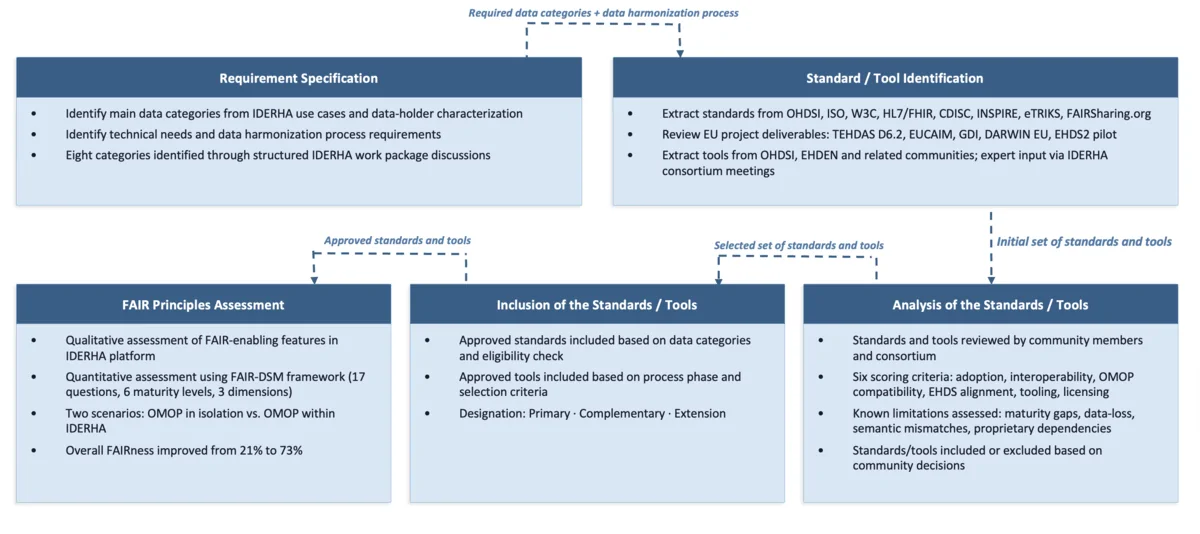
Overview of the data standard and tool assessment process. Identifying data categories and requirements, identifying standards and tools from standard development organizations and European Union (EU) projects. Candidate standards were reviewed in community meetings (excluded/included) and scored on 6 criteria. The approved standards were further assessed through a findable, accessible, interoperable, and reusable (FAIR) assessment process. CDISC: Clinical Data Interchange Standards Consortium; DARWIN: Data Analysis and Real World Interrogation Network; DSM: dataset maturity; EUCAIM: European Federation for Cancer Images; FHIR: Fast Healthcare Interoperability Resources; GDI: Genomic Data Infrastructure; HL7: Health Level Seven; INSPIRE: Infrastructure for Spatial Information in the European Community; ISO: International Organization for Standardization; OHDSI: Observational Health Data Sciences and Informatics; OMOP: Observational Medical Outcomes Partnership; TEHDAS: Towards the European Health Data Space (Joint Action); W3C: World Wide Web Consortium.

### Eligibility Criteria

Standards were considered eligible for evaluation if they met at least one of the following criteria: documented deployment in a comparable EU health data project or initiative; explicit recommendation by TEHDAS or another EHDS-aligned body; active development or endorsement under a recognized international standards organization (ISO, W3C, HL7, OHDSI, Global Alliance for Genomics and Health [GA4GH], or CDISC); or confirmed relevance to one or more of the IDERHA data categories as established through expert input.

### Evaluation Criteria and Scoring

Each eligible standard was evaluated against 6 criteria, each scored qualitatively as high, medium, or low:

Community adoption and maturity: the status of real-world deployment, version stability, and duration of the standard in comparable settings and across EU health data projects.
Technical suitability and interoperability: the degree to which the standard supports the data types, granularity, and exchange mechanisms required by IDERHA, and its compatibility with other selected standards.
(3) OMOP-CDM compatibility: the nature of the standard’s relationship to OMOP-CDM.
EHDS alignment: the extent to which the standard is referenced, recommended, or mandated within the EHDS regulatory framework or TEHDAS recommendations.
Tooling availability and ecosystem support: the availability of open-source or community-supported tools for implementing, validating, and transforming data according to the standard.
Licensing conditions and implementation: whether the standard and its required vocabularies or reference implementations are freely accessible, and the estimated effort required for implementation.

In addition to these scored criteria, each standard was assessed for known limitations and contextual risks, including maturity gaps, documented data loss during conversion, unresolved semantic mismatches, and vocabulary dependencies.

Standards were initially reviewed and discussed within the IDERHA work package meetings described above. Formal scoring was subsequently carried out in separate follow-up meetings by a smaller group of 3 specialists in health data interoperability and standardization, who rated each standard against the 6 criteria through open group discussion, considering the discussions and minutes from the earlier meetings. Where assessments diverged, the group continued discussing until a consensus rating was reached.

### Selection Logic

Where a domain had multiple eligible candidates, the composite score was the number of the 6 criteria rated high; the standard with the highest ratings was designated “primary,” meaning it serves as the main representation format for that data category within IDERHA. If 2 or more candidates had the same number of high ratings, the group kept discussing the standards until they agreed on a final ranking. Standards that provide complementary coverage, serve as source formats requiring conversion to the primary standard, or supply architectural features supporting the primary standard were designated Complementary. Standards under active community development that are not yet production-ready, or whose feasibility within IDERHA has not yet been confirmed, were designated Extension, indicating that they are under evaluation for future adoption.

### Assessment of Adherence to FAIR Principles

To assess the improvement in adherence to FAIR principles achieved through the IDERHA platform, we applied the FAIR-DSM [31] framework to two complementary scenarios: (1) data represented in the OMOP-CDM but existing in isolation (“OMOP in a vacuum”), and (2) the same data implemented within the IDERHA federated platform. The “OMOP in a vacuum” scenario does not represent a real-world situation but serves as a theoretical baseline to quantify IDERHA’s contribution beyond the inherent FAIR-enabling features of OMOP. The IDERHA scenario was likewise scored against the platform’s documented architecture and planned capabilities, rather than through empirical testing of live, operational multisite data exchange.

FAIR-DSM uses 17 questions [32] to evaluate dataset maturity across 3 dimensions—representation and format, content and context, and hosting environment capabilities—and across 6 maturity levels that capture increasing degrees of FAIR implementation:

- Level 0: single-use data (data used once, not shareable, or reusable);
- Level 1: identifiable data (data and basic metadata can be cited and shared);
- Level 2: described data (data are accompanied by structured metadata and documentation);
- Level 3: standardized data (data conform to recognized community or domain standards);
- Level 4: semantically typed data (data and metadata represented with formal semantics enabling interoperability); and
- Level 5: managed assets (enterprise-level governance and asset management).

Each criterion within these levels was evaluated and scored following FAIR-DSM guidance. Results were aggregated as average completion percentages per dimension across levels 1-4, excluding level 5, as IDERHA is a publicly-funded, noncommercial research infrastructure not intended to reach enterprise-grade FAIR governance within the project. Full results are available in Multimedia Appendices 1 and 2.

### Feasibility Assessment and Use Case Demonstration

To evaluate the feasibility of the selected harmonization and quality assessment tools within the IDERHA framework, a pilot feasibility experiment was conducted using synthetic OMOP-CDM data provided by one of the IDERHA data partners, focusing on 3 tools: Achilles and Data Quality Dashboard (DQD) from the OHDSI community, and CDMInspection from the EHDEN community, with the aim of investigating the feasibility of applying these tools to assess the data harmonization process rather than evaluating quantitative results. The experiment proceeded in the following steps: (1) the synthetic dataset was loaded into a local OMOP-CDM instance; (2) each tool was executed against the dataset in its default configuration; (3) tool outputs—including data quality reports, completeness checks, and mapping summaries—were reviewed to assess whether the tools ran successfully, produced interpretable outputs, and identified meaningful data quality issues. To further illustrate the practical application of the selected standards and tools in a real-world context, a personalized lung cancer risk assessment scenario was used as an illustrative use case, demonstrating how heterogeneous data from diverse sources, including hospitals, laboratories, and research institutions, can be harmonized and prepared for federated analysis using the selected standards and tools.

## Results

### Potential Data Sources and Categories

In IDERHA, the main use cases are AI-enabled individualized lung cancer risk assessment, malignancy detection and remote monitoring of patients with lung cancer. Based on these use cases and the data holders that host lung cancer data such as hospitals, cancer registries, or other related databases and programs and on the characterization of their data content, we evaluated the existing datasets available at the data provider sites within IDERHA and analyzed the domains of data they include. We have identified the following main data categories: clinical real-world/routine data derived from electronic health records, imaging, socioeconomic data, environmental data, PREMs and PROMs, molecular biology and genetics, metadata, and security and privacy. Based on these categories, we have selected a set of standards, as indicated in Figure 2, to cover the aforementioned domains. In some categories, multiple standards are presented; in such cases, we have selected one as the main standard for building our data model, while related standards are used either to extend our model or to provide architectural features supporting these standards.

**Figure 2:**
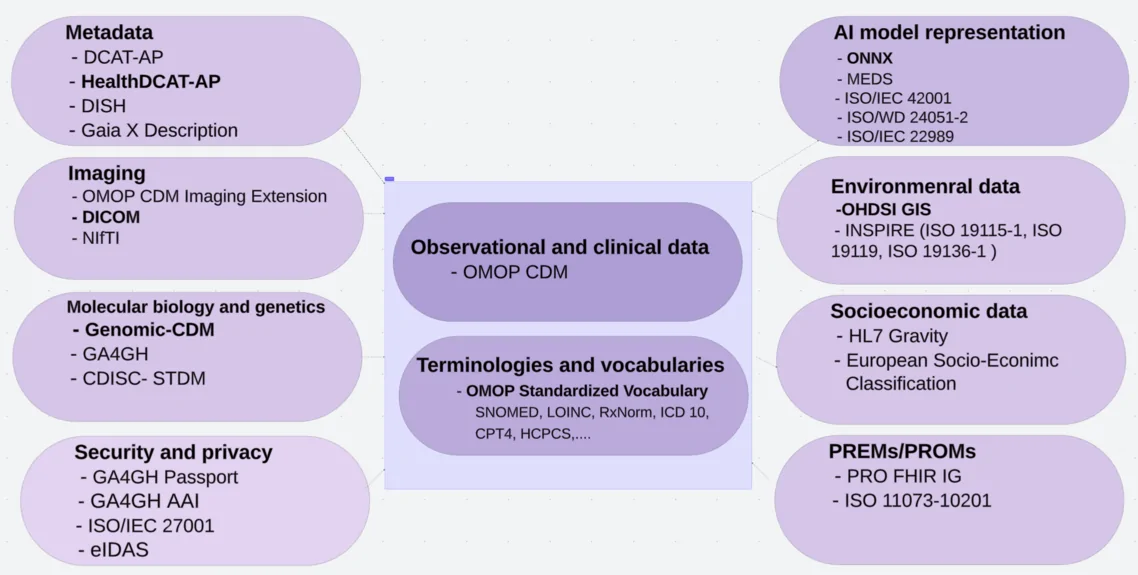
Integration of Heterogeneous Data and Evidence Towards Regulatory and Health Technology Assessment Acceptance selected data standards by category. Observational Medical Outcomes Partnership (OMOP) Common Data Model (CDM) and its standard vocabulary as the core standards; 8 surrounding categories list the candidate standards, with bolded entries marking the primary standard per category. AAI: Authentication and Authorization Infrastructure; CDISC: Clinical Data Interchange Standards Consortium; CDM: Common Data Model; CPT-4: Current Procedural Terminology, 4th Edition; DCAT: Data Catalog Vocabulary; DCAT-AP: DCAT Application Profile for European Data Portals; DICOM: Digital Imaging and Communications in Medicine; eIDAS: Electronic Identification, Authentication, and Trust Services; GA4GH: Global Alliance for Genomics and Health; GIS: geographic information system; HCPCS: Healthcare Common Procedure Coding System; HL7: Health Level Seven; ICD-10: International Classification of Diseases; INSPIRE: Infrastructure for Spatial Information in the European Community; LOINC: Logical Observation Identifiers Names and Codes; MEDS: Medical Event Data Standard; NIfTI: Neuroimaging Informatics Technology Initiative; OHDSI: Observational Health Data Sciences and Informatics; OMOP: Observational Medical Outcomes Partnership; ONNX: Open Neural Network Exchange; PRO: Patient Reported Outcomes; SNOMED CT: Systematized Nomenclature of Medicine-Clinical Terms.

In IDERHA, we have chosen the OMOP-CDM and its standard vocabulary (the bold standards in Figure 2 indicate the main data standard in each category), a well-established framework that provides a shared semantic and structural representation of data among health data providers. OMOP-CDM is considered the primary data standard for harmonizing structured longitudinal electronic health record data. However, the canonical CDM model does not cover all aspects of health data. Considering the scope of the IDERHA project and the need for harmonized data across diverse categories to facilitate analysis and the use of AI algorithms in federated environments, the data can potentially fall into three types: (1) data already in OMOP format (“OMOPed”), (2) data not in OMOP format (eg, FHIR and CDISC), but convertible through existing solutions (as described in the data conformance and harmonization section), and (3) data not fully supported by OMOP-CDM or existing conversion tools, such as economic, environmental, imaging or PREM/PROM data. Currently, we use the OMOP-CDM version 5.4, and considering the OHDSI standard vocabulary, all IDERHA data holders should map their data and internal concepts to it using the proposed data harmonization tools. Given the current conformance status, OMOP-CDM, DICOM are being used for data analysis. The usability of OMOP Imaging, OHDSI GIS, and genomic vocabularies is under discussion in IDERHA, and a feasibility study is underway based on the studies and analysis established in the project. The IDERHA standard collection is available on the FAIRsharing standard collection.

### Overview of the Standards for Secondary Use of Health Data

#### Overview

Based on the assessment described in the Methods section, we identified the main data categories: clinical data and terminologies, imaging, molecular biology and genetics, socioeconomic data, environmental data, PREMs and PROMs, metadata, and security and privacy. For each domain, standards were selected and evaluated for maturity, known limitations, implementation burden, licensing and interoperability. A summary of the evaluation is shown in Table 1, and the extended table including the aforementioned criteria can be found in Multimedia Appendix 3.

**Table 1:** Assessment of health data standards by category for the secondary use of health data in the IDERHA^a^ project. For each of the data categories identified in this study, the table lists the candidate standards evaluated and their scores across 5 criteria each rated high, medium, low, or partial.

Data category and standard		Adoption and popularity	Interoperability	OMOP^b^-CDM^c^ compatibility	EHDS^d^ alignment	Tooling and support	IDERHA role
**Observational** **and** **clinical data**							
	OMOP-CDM v5.4	High	High	Primary	High	High	Primary
	HL7^e^ FHIR^f^	High	High	Partial	High	High	Complementary
	CDISC^g^ SDTM^h^	High	Medium	Partial	Partial	High	Complementary
	CDISC ADaM^i^	High	Medium	Partial	Partial	High	Complementary
**Terminologies and vocabularies**							
	OMOP standardized vocabularies	High	High	Primary	High	High	Primary
	SNOMED CT^j^	High	High	Primary	High	High	Complementary
	LOINC^k^	High	High	Primary	High	High	Complementary
	RxNorm/ATC^l^/*ICD-10*^m^/CPT-4^n^/HCPCS^o^	High	High	Primary	High	High	Complementary
**Metadata**							
	DCAT-AP^p^	Medium	High	Not applicable	High	Medium	Primary
	HealthDCAT-AP	Low	High	Complementary	High	Low	Primary
	Gaia-X Self-Description	Medium	High	Not applicable	High	Medium	Complementary
	CDISC Define-XML	High	Medium	Partial	Partial	High	Complementary
**Imaging**							
	DICOM^q^	High	High	Extension	High	High	Primary
	OMOP-CDM Imaging extension	Low	Medium	Extension	Partial	Low	Extension
	NIfTI^r^	Medium	Medium	Partial	Partial	Medium	Complementary
	Genomic CDM (OHDSI^s^ Oncology WG)	Low	Medium	Extension	Partial	Low	Extension
**Molecular biology and genetics**							
	GA4GH^t^	High	High	Complementary	Partial	High	Complementary
	CDISC SDTM	High	Medium	Partial	Partial	High	Complementary
**Security and privacy**							
	GA4GH Passport	Medium	High	Not applicable	Partial	Medium	Primary
	GA4GH AAI^u^	Medium	High	Not applicable	Partial	Medium	Primary
	ISO/IEC^v^ 27001:2022	High	Medium	Not applicable	High	Medium	Complementary
	eIDAS^w^	High	High	Not applicable	High	Medium	Primary
**AI model representation**							
	ONNX^x^	High	High	Not applicable	Partial	High	Primary
	MEDS^y^	Low	Medium	Partial	Partial	Low	Complementary
	ISO/IEC 42001:2023	Medium	Not applicable	Not applicable	Partial	Low	Complementary
	ISO/WD^z^ 24051-2	Low	Low	Not applicable	Partial	Low	Complementary
	ISO/IEC 22989	Medium	Not applicable	Not applicable	Partial	Low	Complementary
**Environmental data**							
	OHDSI GIS^aa^ Extension	Low	Medium	Extension	Partial	Low	Extension
	INSPIRE^ab^ + ISO 19115-1/ISO 19119/ISO 19136	High	Medium	Complementary	High	Medium	Complementary
**Socioeconomic data**							
	HL7 FHIR Gravity	Medium	Medium	Partial	Partial	Medium	Complementary
	ESeC	Medium	Low	Partial	Partial	Low	Complementary
**PREMs^ac^/PROMS^ad^**							
	PRO^ae^ FHIR IG^af^	Medium	High	Partial	Partial	Medium	Primary
	ISO/IEEE^ag^ 11073-10201:2020	Medium	Medium	Partial	Partial	Low	Complementary

^a^IDERHA: Integration of Heterogeneous Data and Evidence Towards Regulatory and Health Technology Assessment Acceptance.

^b^OMOP: Observational Medical Outcomes Partnership.

^c^CDM: Common Data Model.

^d^EHDS: European Health Data Space.

^e^HL7: Health Level Seven.

^f^FHIR: Fast Healthcare Interoperability Resources.

^g^CDISC: Clinical Data Interchange Standards Consortium.

^h^SDTM: Study Data Tabulation Model.

^i^ADaM: Analysis Data Model.

^j^SNOMED CT: Systematized Nomenclature of Medicine-Clinical Terms.

^k^LOINC: Logical Observation Identifiers Names and Codes.

^l^ATC: Anatomical Therapeutic Chemical.

^m^ICD-10: International Classification of Diseases.

^n^CPT-4: Current Procedural Terminology, 4th edition.

^o^HCPCS: Healthcare Common Procedure Coding System.

^p^DCAT-AP: Data Catalog Vocabulary Application Profile for European Data Portals.

^q^DICOM: Digital Imaging and Communications in Medicine.

^r^NIfTI: Neuroimaging Informatics Technology Initiative.

^s^OHDSI: Observational Health Data Sciences and Informatics.

^t^GA4GH: Global Alliance for Genomics and Health.

^u^AAI: Authentication and Authorization Infrastructure.

^v^IEC: International Electrotechnical Commission.

^w^eIDAS: electronic Identification, Authentication, and Trust Services.

^x^ONNX: Open Neural Network Exchange.

^y^MEDS: Medical Event Data Standard.

^z^ISO/WD: ISO/Working Draft.

^aa^GIS: Geographic Information System.

^ab^INSPIRE: Infrastructure for Spatial Information in the European Community.

^ac^PREM: patient-reported experience measure.

^ad^PROM: patient-reported outcome measure.

^ae^PRO: patient reported outcome.

^af^IG: implementation guide.

^ag^IEEE: Institute of Electrical and Electronics Engineers.

#### Clinical Data and Terminologies

OMOP-CDM is a well-established research data model used across major European health data initiatives including DARWIN EU, EHDEN, EUCAIM, and OPTIMA [19]. OMOP was chosen as the primary standard for federated analysis because it serves a different purpose than FHIR. While FHIR is designed for the real-time exchange of patient data between clinical systems, the OMOP-CDM is specifically built to support research queries across large observational datasets, which aligns with the IDERHA use cases. OMOP also comes with a standard set of analysis tools that allow all sites to run the same queries without sharing raw patient data, which is critical for privacy-preserving federated research. However, converting source data into OMOP (the extract, transform, and load [ETL] process) is technically challenging and needs ongoing maintenance. Clinical concepts that do not have a direct OMOP equivalent have to be approximated, which can introduce errors in analysis. OMOP’s coverage of imaging, genomics, patient-reported outcomes, and environmental data is incomplete and relies on community extensions that have not yet been widely tested across multiple sites. Sites also need specialist staff to work with OMOP tools, which can slow down onboarding [33-35].

FHIR is widely used across EU health data projects and is the dominant format for electronic health record exchange. It is a well-supported standard with strong tooling, and a number of tools can convert FHIR data into OMOP-CDM format. In IDERHA, FHIR is treated as an input format—data arriving in FHIR is converted to OMOP before analysis, rather than used directly. However, converting FHIR to OMOP can result in some data being lost, especially for clinical concepts that do not map cleanly between the 2 formats. There might also be some interoperability issues since EU member states have implemented FHIR differently, so FHIR data from one country may not be directly comparable with FHIR data from another, even though both technically comply with the standard.

CDISC Study Data Tabulation Model (SDTM) [36] is the required format for submitting clinical trial data according to FDA guidelines and is recommended by the EMA [17]. It is a mature and well-supported standard in the regulatory context. However, it was designed for submitting data to regulators, not for running research analyses—it does not capture the longitudinal, time-ordered information that observational research needs. Converting SDTM to OMOP also may result in data loss because not all parts of SDTM have validated OMOP equivalents. CDISC Analysis Data Model (ADaM) [37], which is used alongside SDTM for analysis datasets in regulatory submissions, includes derived variables that do not have direct equivalents in OMOP and require manual mapping.

Standardized vocabularies [38,39] are key parts of OMOP standards that help integrating and querying health data among different sources of data. It provides a uniform semantic representation of data and stores metadata about vocabularies, concepts, and their corresponding domain. There may also be nonstandard concepts in the data sources, called “source concepts.” Nonstandard concepts are linked to standard concepts through a mapping process. Their main limitation is that coverage is uneven: rare diseases, social economic factors, and patient-generated data are poorly represented. Concepts without a good match in the vocabulary may be left unmapped or approximated inconsistently across sites. Individual vocabularies also have their own constraints: Systematized Nomenclature of Medicine-Clinical Terms (SNOMED CT) requires a license (free for most EU countries); Logical Observation Identifiers Names and Codes (LOINC) is free but does not fully cover European lab tests or patient outcome measures; RxNorm is US-focused and often needs an extra mapping step to work with European drug codes. Current Procedural Terminology, 4th Edition (CPT-4) and Healthcare Common Procedure Coding System require a Unified Medical Language System (UMLS) account, which is a barrier for some sites.

### Health Care Metadata Standards

Different standard models have been established to facilitate the description and aggregation of metadata within federated infrastructures [26,40]. The DCAT [41] is a Resource Description Framework–based vocabulary that enhances the interoperability among data catalogs. The DCAT-AP [8] is another standard that extends DCAT and is primarily designed to improve cross-portal metadata descriptions and searches to increase the discoverability of data across EU members. The main problem is that DCAT-AP was built for general public data, not health data—it lacks fields for describing clinical data types, consent requirements, patient populations and data quality. As part of the EHDS goals, the HealthDCAT-AP [42] standard extends the DCAT standards by adding classes, properties and changing some cardinalities to support health care–specific requirements. It is a required standard under the EHDS regulation, and it directly addresses the main gap in DCAT-AP for health data. The main risk is that it is still being developed: key definitions and controlled vocabulary lists are not yet finalized, so adopting it too early could mean rework later. HealthDCAT-AP is considered the main standard for implementing metadata at IDERHA.

Gaia-X Self-Descriptions [43] provide machine-readable information about data services and are designed for automated service discovery in federated data spaces and are considered for extending the IDERHA metadata model. Their limitation is that they describe services and providers rather than clinical datasets, so they cannot replace DCAT-AP or HealthDCAT-AP for dataset-level discovery. CDISC Define-XML [44] complements SDTM and ADaM standards in terms of metadata submissions. It could be used as a reference document during data onboarding: it describes the structure and content of incoming clinical trial datasets, which helps to document the data provenance.

### Representation of AI Models

Open Neural Network Exchange (ONNX) [45] is an open format for representing ML models that works across all major AI frameworks. This broad compatibility is the main reason for choosing it: it allows AI models trained at one site to be shared and run at other sites regardless of the framework used locally. There is no comparable alternative that is both as widely supported and freely available (MIT license). The main limitations are that ONNX does not record how a model was trained or how it performs, that some advanced or custom model architectures may not convert correctly, and that different versions of ONNX can sometimes be incompatible with each other.

Medical Event Data Standard [46] is a newer format for structuring medical record data specifically for training large AI models. It is included in IDERHA as a supporting standard for AI pipelines. However, it has limited adoption and immature and it is free (MIT license). The ISO AI standards—ISO/IEC 42001:2023 [47] for AI management systems, and ISO/IEC 22989 [48] for AI terminology—are used for governance and documentation purposes. They are useful for aligning with EU AI Act compliance requirements, but they are management standards, not technical ones, they do not provide tools, methods, or implementation guidance.

### Imaging Data

DICOM [49] is the established global standard for medical imaging and is built in most imaging devices on the market. The main practical limitations for federated research are that DICOM metadata quality varies significantly between institutions—some sites tag their DICOM files inconsistently or incompletely—which makes automated federated search and AI model training harder. Device manufacturers also add custom DICOM extensions that can reduce compatibility between sites, and adoption in digital pathology is still incomplete. The OMOP-CDM Imaging Extension was developed to connect DICOM imaging data to the OMOP research framework by adding new tables for image records and features. The problem is that it is not mature enough for reliable multisite use yet: it is not part of the main OMOP-CDM release, the standard OMOP tools do not cover it, and vocabulary mappings are incomplete for many imaging types. Neuroimaging Informatics Technology Initiative (NIfTI) [50] is the standard format in academic magnetic resonance imaging and brain imaging research. Its weakness is that it does not fully store information related to patient identifiers, scan settings, and provenance.

### Molecular Biology and Genetic Data

The SDTM [51], which was developed by CDISC [52], standardizes clinical data in tabular format. The GA4GH [53,54] maintains and improves standard file formats for genomic data, such as SAM (Sequence Alignment Map), BAM (Binary Alignment Map), CRAM (Compressed Reference-Aligned Map), and VCF (Variant Call Format)/BCF (Binary Call Format). It also works on representing and interpreting individual genetic variants (using formats like VRS [Variation Representation Specification] and VA [Variant Annotation]) and sharing individual health data through phenopackets. The key limitation is that GA4GH does not provide a clinical data model—it handles the genomic data files but cannot on its own link genomic findings to clinical outcomes. It needs to be used alongside a clinical data model. Beacon v2 [55] adoption is also uneven across European sites, and using Phenopackets to encode clinical context requires structured clinical data that not all sites may have available. All GA4GH standards are free and open source.

The OMOP Genomic CDM [12], developed by the OHDSI Oncology Working Group, extends OMOP-CDM to include next-generation sequencing data, with new tables for sequencing results and vocabularies for genes (HGNC) [56] and variants (Human Genome Variation Society). However, it is not yet mature: the tables are not part of the official OMOP-CDM release, variant representation requires specialized bioinformatics tools, and there are no validated multisite deployments to draw on. It remains an extension under consideration rather than a deployed standard.

### Socioeconomics Data

One of the pioneering projects in managing socioeconomic data is the HL7 FHIR Gravity Project [57], which created a framework for sharing information on social factors that affect health (called Social Determinants of Health [SDOH]) [58]. This standard is used in clinical activities like screening, diagnosis, setting treatment goals, and treatment. The project focuses on improving how different systems work together to address national socioeconomic issues. The main problem for the European context is that Gravity was built around US social service structures, terminology, and classification systems. EU socioeconomic categories, housing definitions, and social care frameworks are different, and are not well represented. There is also no validated pathway for converting Gravity FHIR data into OMOP-CDM, so custom mapping would be required. EU has made many efforts to integrate socioeconomic data for advancing research by analysis of quality of life and living conditions parameters. The European Socio-Economic Classification (ESeC), developed by the EU’s Sixth Framework Programme Priority 7 (Citizens and Governance in a Knowledge-Based Society), is used for categorizing occupations and employment status. The fundamental limitation is that ESeC is only a classification scheme—it has no data exchange format, no API, and no machine-readable structure. Mapping national occupational data to ESeC requires manual work and specialist knowledge. In OMOP-CDM, socioeconomic data can be partially standardized using related observation standard vocabularies; however, it does not provide a comprehensive data structure for representing socioeconomic data [59].

### Medical Device Data

IEEE standards for medical device communication, such as the ISO/IEEE 11073 [60] family, provide a common, machine-readable structure and terminology for capturing measurements, device states, and alarms at the point of care. The medical device data can be represented in OMOP-CDM or large-scale, reproducible research and federated analytics, using tables such as Measurement for numeric values and Device_Exposure. The main limitation is that the standard is mostly built into specific device firmware and few open tools implement it. Consumer wearables, which are increasingly important for remote monitoring, typically use their own protocols and require custom adaptors. There is also no standardized OMOP ETL pipeline for wearable data streams, meaning each site would need to build its own conversion process.

### Environmental Data

Currently, the EU establishes the INSPIRE [61] to provide guidelines and instructions on how to manage environmental and spatial data for EU members to improve interoperability and data services. The INSPIRE guidelines are based on the technical specifications of ISO geographic information related standards, including ISO 19115-1:2014 [62] Geographic Information Metadata, ISO 19119:2016 [63] Geographic Information Services, and ISO 19136-1:2020 [64] Geographic Information Geography Markup Language.

The OHDSI GIS expands the OMOP-CDM by adding geospatial data and patient environmental exposure histories, as well as patient locations. It also provides a data structure for storing spatial, environmental, behavioral, socioeconomic, phenotypic, and toxin-related determinants. It includes related vocabularies, including the Exposome and SDOH vocabularies. The key advantage of OHDSI GIS over using INSPIRE data is that it allows environmental exposure histories to be queried alongside clinical outcomes within the same OMOP analytics framework. However, the extension is at an early stage and its tables, vocabularies and tools are not yet finalized.

### PREMs and PROMs Data

For addressing the PREMs and PROMs data, currently, the FHIR Patient Reported Outcomes (PRO) [65] project is being considered. The data provided by medical devices and wearable gadgets should comply with the ISO/IEEE 11073-10701-2022 [60] which is used for point-of-care medical device Communication Metric Provisioning by Participants in Service-Oriented Device Connectivity Systems. The International Consortium for Health Outcomes Measurement (ICHOM) has published a collection of patient-centered outcome measures for tracking patients with lung cancer along with relevant demographic, clinical and tumor case-mix variables for risk adjustments [66].

### Security and Privacy

The EU Regulation on Electronic Identification, Authentication, and Trust Services (eIDAS) [67] addresses electronic identification (eID) and improves the use of trust services and eID across EU members. Its main limitation in practice is that cross-border eID interoperability is inconsistent. Partners outside the EU are also not covered by eIDAS and need separate identity arrangements. GA4GH Passports and the Authentication and Authorization Infrastructure (AAI) are used alongside eIDAS in IDERHA to manage access to specific datasets within the federated infrastructure. Interoperability between different Passport broker implementations has also not been fully tested at scale, and managing tokens for long-running federated analyses adds complexity.

### IDERHA Data Conformance and Harmonization

To support data harmonization, we assembled a set of tools drawn mainly from the OHDSI community, with additional tools from other communities where appropriate. We organized them by function: data transformation/ETL, data characterization and quality assessment, and vocabulary/metadata management. Based on our initial review of IDERHA data holders and data types—and recognizing that data may appear in multiple standard formats—we placed particular emphasis on transformation tools, including pipelines for FHIR-to-OMOP-CDM [68] (HL7 FHIR Accelerator Vulcan) and CDISC SDTM-to-OMOP-CDM [69] conversions and Image CDM, to produce a unified OMOP-CDM dataset. The data extraction and loading are conducted with the support of OHDSI White Rabbit [70], Rabbit-in-a-Hat [70] to scan and map the data. Usagi [71] is considered to facilitate mapping by term similarity assessment. Athena [72] and ontology services [73] are used to manage the controlled vocabularies and terminologies. However, tools such as Open Refine [74], and Pentaho Kettle [75] are also used for mapping source concepts to OMOP-CDM. For data characterization and quality assessment, we use the OHDSI R packages Achilles [76] and DQD [77]. Additionally, as part of the data-harmonization assessment, we use the CDMInspection [78] package to evaluate the quality of mapped vocabularies and to produce technical infrastructure reports; this tool is part of the EHDEN community toolkit (Figure 3).

**Figure 3:**
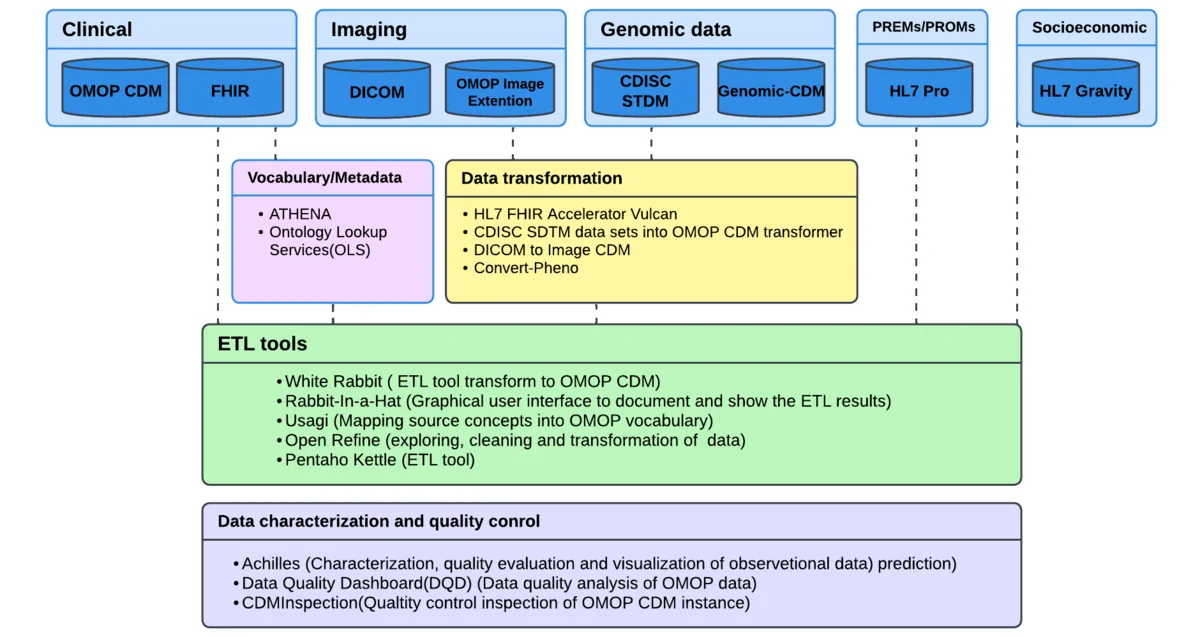
Integration of Heterogeneous Data and Evidence Towards Regulatory and Health Technology Assessment Acceptance conformance and harmonization tools across the data-preparation pipeline. Source data from each category goes through vocabulary/metadata harmonization or format transformation as needed, brought together in a common extract, transform, and load (ETL) stage, and then passed through a final data characterization and quality-control step before use. CDISC: Clinical Data Interchange Standards Consortium; CDM: Common Data Model; DICOM: Digital Imaging and Communications in Medicine; FHIR: Fast Healthcare Interoperability Resources; HL7: Health Level Seven; OMOP: Observational Medical Outcomes Partnership.

Converting source data into OMOP-CDM from other standards presents several technical and semantical challenges. The extent of data loss depends significantly on the characteristics of the source data and its adherence to standard vocabularies [79]. There have been limited experiments that have performed SDTM-to-OMOP conversion and lack established guidelines; real-world implementations require complex data imputation strategies. MedDRA—the main terminology used for adverse events in clinical trials—has no direct links to OMOP standard concepts, so mapping requires a combination of automated matching and extensive manual expert review to produce usable concept maps. Medication data encoded using WHODrug, a vocabulary not yet part of OMOP-CDM, needs to be handled through custom mapping. For complex or rare diseases, standard OMOP vocabularies do not cover all disease subtypes and study-specific concepts, requiring the creation of custom concepts that are then incorporated into the concept hierarchy to remain accessible in analytics. Overall, converting SDTM to OMOP is not a straightforward process and requires significant manual effort, domain expertise, and custom solutions to reduce data loss and preserve the meaning of the source data [80]. FHIR-to-OMOP conversion has received more attention compared to SDTM-to-OMOP, with several studies and international initiatives such as the HL7 Vulcan project and the All of Us Research Program having explored and demonstrated its feasibility at scale [81]. Despite this, several challenges remain. FHIR is designed for exchanging patient data between clinical systems, while OMOP is built for research and analysis, and this difference in purpose means that not all FHIR data fits neatly into OMOP tables, and some information is inevitably lost during the conversion. Although both standards use common vocabularies such as SNOMED CT, LOINC and RxNorm, Codes that are specific to certain hospital systems or vendors, often have no match in standard OMOP vocabularies, leading to mapping. Converting data back from OMOP to FHIR is also limited, as patient-level details removed during the conversion to OMOP for privacy reasons cannot be recovered, making the reverse conversion only useful for testing and validation [82].

### Sample Use Case Scenario: Personalized Lung Cancer Assessment

The first use case that IDERHA aims to conduct during the Innovative Health Initiative (IHI) funding period is the personalized lung cancer assessment based on patient data from different heterogeneous entities and datasets, including hospitals, laboratories and research institutions. The collected data from diverse data sources are in different common data formats including FHIR, HL7 V3, DICOM, CDISC SDTM, and other proprietary internal data models. This variability presents challenges in ensuring interoperability and consistency across datasets. To address these challenges, data components that align with the OMOP-CDM undergo a systematic transformation into their corresponding OMOP representations. For instance, FHIR data can be converted to OMOP-CDM using HL7 Vulcan, which facilitates the integration of health care data across different systems. Imaging data are adapted to fit the OMOP Imaging extension, allowing for a standardized approach to managing imaging-related information. Additionally, CDISC SDTM data are transformed into OMOP format using a specialized CDISC to OMOP converter.

The datasets that lack a corresponding OMOP data model, for instance, socioeconomic data, are transmitted through the HL7 Gravity data standard, or partially implemented through OMOP standard vocabularies. Furthermore, patient-reported outcomes collected via citizen applications are shared through HL7 FHIR PRO protocols or are in OMOP-CDM in advance, enabling patients to contribute valuable insights regarding their health experiences. Each segment of data from patient with lung cancer may originate from any of the aforementioned sources, and these datasets are made accessible through the federated nodes of the IDERHA platform. Each federated node possesses the capability to share metadata about its datasets by the DCAT standard. Although the main standard for building our metadata model is DCAT, the model will further be extended based on HealhDCAT-AP and Gaia-X Description. This shared metadata is aggregated and managed by the centralized node of IDERHA, which plays a crucial role in facilitating federated AI processing. Data harmonization and quality control tools serve both each federated node and the central node in terms of data curation and integration of different OMOP vocabulary and terminologies and data quality control. Figure 4 demonstrates the diverse data sources and potential centers that function as repositories for information from patients with lung cancer, emphasizing the interconnectivity of these entities in fostering a comprehensive understanding of lung cancer data within the IDERHA framework. To prepare for the ETL of longitudinal health care databases into the OMOP-CDM, White Rabbit can be applied to scan the data and generate a report with all the necessary information to start designing the ETL. Its primary function is to examine the source data, providing detailed insights into the tables, fields, and values present. This report can serve as a reference for designing the ETL, especially when used alongside the Rabbit-In-a-Hat tool. Unlike standard data profiling tools, White Rabbit aims to prevent the display of personally identifiable information in the generated output file. Each federated node or the central node can use the OHDSI Usagi (or tools such as Pentaho kettle) to assist in the manual creation of code mappings. It offers suggested mappings based on the textual similarity of code descriptions. If the automated suggestions are inaccurate, users can search for the correct target concepts. Ultimately, users can approve mappings for use in the ETL process. Users can use the OHDSI Achilles tool at each federated node or the central node for the characterization, quality assessment, and visualization of observational health databases based on the OMOP-CDM. Meanwhile, the DQD applies a standardized data quality assessment terminology to data formatted in the OMOP-CDM. While Achilles conducts characterization analyses to provide a comprehensive visual understanding of a CDM instance, the DQD examines data table by table and field by field to quantify the number of records in the CDM that do not meet specified criteria. The data characterization and data quality reports are used for the assessment and monitoring of data holders in the IDERHA community.

**Figure 4:**
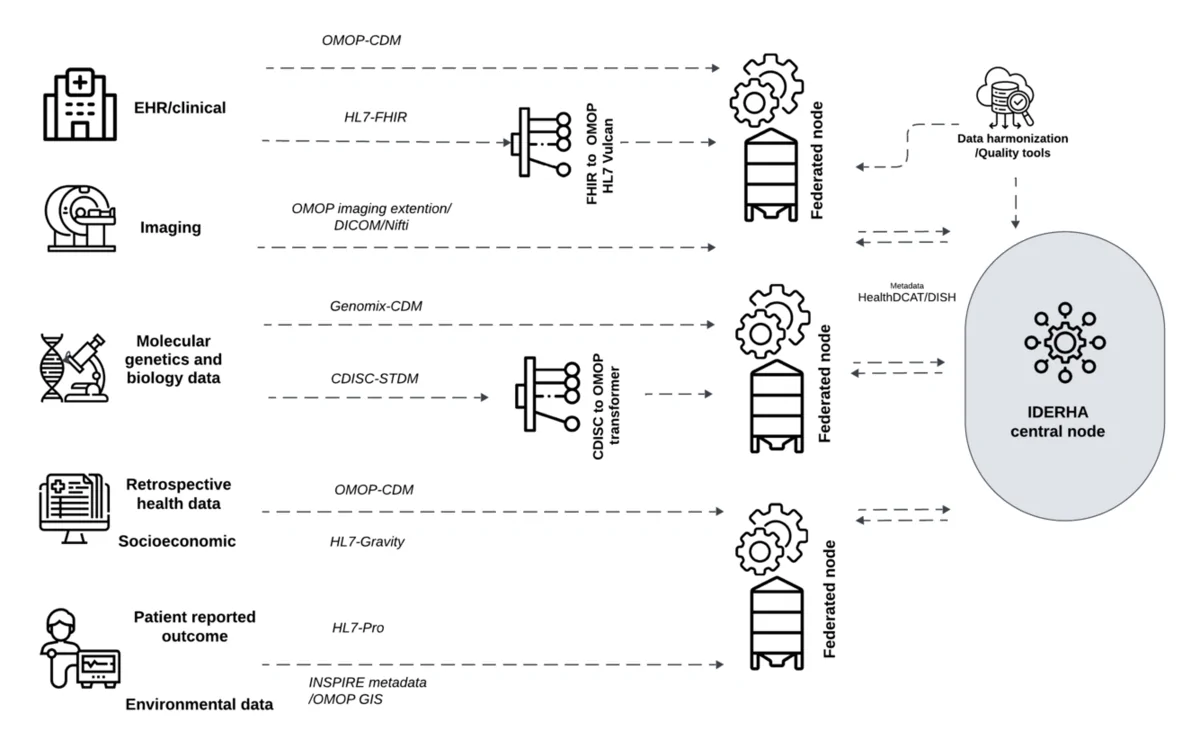
Integration of Heterogeneous Data and Evidence Towards Regulatory and Health Technology Assessment Acceptance (IDERHA) potential data sources, flow, and conversion to Observational Medical Outcomes Partnership (OMOP) Common Data Model (CDM) in a sample federated lung cancer risk assessment scenario. The data from heterogeneous data sources are converted to OMOP-CDM at each federated node, which exchanges analysis results and metadata with the IDERHA central node. DICOM: Digital Imaging and Communications in Medicine; EHR: electronic health record; FHIR: Fast Healthcare Interoperability Resources; GIS: geographic information system; HL7: Health Level Seven; INSPIRE: Infrastructure for Spatial Information in the European Community; NIfTI: Neuroimaging Informatics Technology Initiative.

### Data Harmonization Experiment: Data Characterization and Quality Check on Synthetic Data

We conducted a feasibility study on parts of the proposed data harmonization tools to assess their suitability for evaluating the harmonization process on synthetic data provided by one of our data partners. We ran the Achilles and DQD packages from the OHDSI community and the CDMInspection package from the EHDEN community. The synthetic dataset was extracted from their OMOP database, for which the original mapping and ETL were performed using WhiteRabbit, Rabbit in a Hat, and Usagi. However, due to the initial data-minimization agreement, the synthetic data did not include certain OMOP-CDM entities, specifically Observation, Measurement, and Device and was in CSV format. We imported the synthetic data into our PostgreSQL OMOP-CDM v5.4 schema, built for data harmonization using ETLSyntheaBuilder. The OMOP vocabularies were imported from Athena and supplemented with CPT-4 using a UMLS API key (v5.0, February 27, 2025). The aim of this experiment was not to evaluate quantitative results, but to investigate the feasibility of using these tools to assess the data-harmonization process. A summary of the extracted results is provided in Multimedia Appendix 4. By running the Achilles tool, we examined summary statistics, including the number of patients, gender distribution, study-year range, and age. Using the DQD package, we reported the percentages of passed and failed data-quality checks and summarized the main data-quality domains—conformance, completeness, and plausibility. Additionally, we describe the variable mappings, data-source metadata, and the existing technical OHDSI packages using the CDMInspection tool. The results of these 3 tools together provide an initial indication of the technical feasibility of running the selected toolset within the IDERHA infrastructure specifically while the onboarding of the new data holders. However, the scope of this experiment is limited: the synthetic data excluded some OMOP-CDM tables—Observation, Measurement, and Device. Consequently, it should be further tested on real-world clinical data in the next steps.

### IDERHA’s Compliance With the FAIR Principles

#### Overview

One of IDERHA’s core objectives is to provide a data platform that adheres to the FAIR principles, which are essential for enabling federated ML in health care. In such an environment, FAIR compliance facilitates efficient data accessibility, integration, and secondary use, thereby unlocking the full potential of AI-driven health care research. A foundational step toward this goal is IDERHA’s adoption of established health data standards. By building upon the OMOP-CDM and its globally standardized vocabularies and concepts, IDERHA ensures a harmonized representation of clinical and observational health data as a basis for interoperability and data reuse.

While OMOP provides a strong FAIR-aligned foundation, achieving full FAIR compliance in practice requires more than adherence to data standards—it also depends on infrastructure, governance, and metadata management. In the following, we outline how the use of the OMOP-CDM establishes a baseline level of adherence to FAIR principles and how the IDERHA platform substantially builds upon this foundation. To do this, we combine a qualitative description of FAIR-enhancing features with a quantitative evaluation of FAIR maturity using the FAIR-DSM [31] framework. This integrated approach demonstrates how IDERHA advances OMOP data from standardized datasets toward fully FAIR, discoverable, and reusable digital assets within a federated research ecosystem.

#### Qualitative Assessment of FAIR-Enabling Features in IDERHA

##### Findability

OMOP contributes to findability through its standardized vocabularies, globally unique concept identifiers, and consistent data structure, which together enable coherent indexing and cross-site referencing of health information. Building on this foundation, IDERHA extends findability to the platform level by adopting metadata standards such as HealthDCAT-AP. It maintains comprehensive catalogs of datasets, metadata, and algorithms, each assigned a globally unique and persistent identifier. This integrated approach allows resources to be effectively registered, indexed, and discovered across the federated network, ensuring full traceability and searchability within and beyond the IDERHA ecosystem.

##### Accessibility

OMOP facilitates accessibility by providing a standardized data structure and a shared ecosystem of tools that enable consistent querying and analysis across institutions. Building on this foundation, IDERHA integrates secure and compliant access mechanisms tailored to federated health data environments. Through solutions such as eIDAS and AAI for authentication and authorization, and a governance framework aligned with General Data Protection Regulation and national regulations, IDERHA ensures that data can be accessed responsibly and transparently across the platform while maintaining the highest standards of privacy and data protection.

##### Interoperability

OMOP establishes strong interoperability within the clinical and observational health data domain through its standardized data structures, harmonized vocabularies, and community-driven conventions. Building on this robust foundation, IDERHA extends interoperability beyond the clinical scope by adopting standardized metadata models such as DCAT and Health DCAT-AP and by selecting dedicated tools for aligning and mapping non-OMOP data sources, including patient-generated data and other health-related domains as described in this work. A platform-wide strategy ensures consistent use and documentation of OMOP extensions, promoting semantic coherence and interoperability across diverse datasets and research contexts within the federated IDERHA environment.

##### Reusability

OMOP promotes reusability through its standardized data structures, harmonized vocabularies, and an active international community that fosters transparent documentation and shared best practices. Building on this foundation, IDERHA enhances reusability by adopting the HealthDCAT metadata standard, which captures detailed information on data provenance, licensing, and harmonization workflows such as ETL processes. These metadata fields make datasets and their context more transparent and support reuse across the federated platform. While certain licensing constraints from proprietary vocabularies remain outside the project’s scope, IDERHA’s structured approach to metadata management substantially advances the clarity, traceability, and responsible reuse of health data.

Thus, to operationalize FAIR principles, IDERHA integrates tooling for metadata capture (eg, DCAT-AP compliant profiles), provenance tracking (via HealthDCAT), and vocabulary linkage through OHDSI vocabularies and mappings (Table 2) [83].

**Table 2:** IDERHA’s^a^ actions to enhance adherence to findable, accessible, interoperable, and reusable (FAIR) principles. The table maps concrete IDERHA design choices to the FAIR principles, organized by principle.

FAIR principle and aspect of FAIR			IDERHA’s actions
**Findable**			
	Enforced standardized metadata	IDERHA recommends and uses metadata standards such as (Health)DCAT^b^	
	Searchability across repositories	IDERHA will provide data and algorithm catalogs with rich metadata for effective searches	
	Unique identifiers and URIs for datasets, data services, and data providers	Datasets, data services, data providers within the IDERHA catalog will have unique IDs and URIs	
**Accessible**			
	Authentication/authorization mechanism	IDERHA uses authentication and authorization services and infrastructures such as eIDAS^c^ and AAI^d^	
	Data sensitivity	IDERHA architecture ensures that all data can be accessed only in compliance with GDPR^e^ and other applying laws/guidelines	
**Interoperable**			
	Domain-specific focus	IDERHA defines data standards for data from domains not covered by OMOP^f^; a comprehensive data model for IDERHA is still under development	
	Enforced standardized metadata	IDERHA recommends and uses metadata standards such as (Health)DCAT	
	Consistency with local extensions	Currently ongoing work	
**Reusable**			
	Provenance and context	IDERHA’s metadata standard, (Health)DCAT, encompasses data versioning and source information. While HealthDCAT includes ETL^g^ processes in text format, it currently lacks support for a machine-readable format	
	License and usage information	IDERHA’s metadata standard (Health)DCAT covers license and usage information	
	Dependence on licensed standards	Currently not addressed	

^a^IDERHA: Integration of Heterogeneous Data and Evidence Towards Regulatory and Health Technology Assessment Acceptance.

^b^DCAT: Data Catalog Vocabulary.

^c^eIDAS: Electronic Identification, Authentication, and Trust Services.

^d^AAI: Authentication and Authorization Infrastructure.

^e^GDPR: General Data Protection Regulation.

^f^OMOP: Observational Medical Outcomes Partnership.

^g^ETL: extract, transform, and load.

#### Quantitative Assessment of FAIR Maturity Using FAIR-DSM

The improvement in adherence to FAIR principles achieved through IDERHA was assessed using the FAIR-DSM framework (see “Methods” section for more details).

The FAIR-DSM evaluation shows a substantial and quantifiable increase in FAIR maturity when OMOP data are embedded in the IDERHA planned ecosystem. Across the first 4 maturity levels (excluding level 5), the average adherence to FAIR principles increased from 42% to 82% in *Representation* and *Format*, 22% to 66% in *Content* and *Context*, and 0% to 72% in *Hosting Environment Capabilities*, resulting in an overall improvement from roughly 21% to 73% adherence to FAIR principles. Level 1 criteria are now fully fulfilled, levels 2-3 are largely met, and approximately one-third of level 4 criteria are achieved, reflecting emerging cross-study interoperability within the federated environment.

The most pronounced improvements were observed in the metadata and hosting environment dimensions, where IDERHA adds the greatest value beyond OMOP’s structural standardization. Through its metadata catalog and federated infrastructure, IDERHA is designed to ensure that data are not only standardized but also discoverable, accessible, and governed in a compliant and transparent manner across the consortium.

While any data platform naturally enhances hosting capabilities compared to isolated data, IDERHA will go significantly beyond a conventional hosting solution by embedding FAIR-aligned metadata, persistent identification, and cross-node discovery mechanisms into its architecture. This elevates OMOP data from standardized datasets to interoperable and reusable digital assets suitable for federated analysis within the planned IDERHA ecosystem.

Taken together, these results point to a major increase in FAIR maturity across all dimensions once the IDERHA platform is fully implemented and operational. The few remaining unmet FAIR-DSM criteria relate to advanced semantic capabilities—such as full linked data representation and the definition of common data elements—which are beyond the project’s current technical scope. The complete FAIR-DSM results for both scenarios, including all unfulfilled criteria, are provided in Multimedia Appendices 1 and 2.

## Discussion

Currently, the EHDS Regulation establishes high-level rules on data governance, access, and interoperability for the exchange and reuse of electronic health data. However, it does not provide detailed guidance on standardizing all aspects of health data or on the time-consuming, complex process of data harmonization. This paper presents the IDERHA roadmap for adopting a set of standards and tools tailored to the secondary use of health data, and it provides an overview of the relevant standards, principles, and harmonization tools. The selection of standards for each data category considered well-established specifications from various communities and EU organizations, as well as IDERHA’s needs and aims, and was refined through extensive discussions with project members. This approach helps ensure that the adopted standards are both relevant and effective for achieving IDERHA’s objectives. The selected standards and transformation tools—and how they are used within IDERHA—are demonstrated through a use-case scenario and a feasibility study using OHDSI and EHDEN tools. These tools are not mandatory; teams may use local alternatives if they follow the same logic and maintain documented quality checks.

These state-of-the-art standards and associated technical requirements would facilitate both federated data processing approaches and the application of the FAIR principles [67] throughout the project. Consequently, the interoperability and reusability of the system will improve. Therefore, we assessed the inherent adherence to FAIR principles of our selected data standards using the example of our main standard, OMOP-CDM. While OMOP-CDM covers many important FAIR aspects, particularly enhancing interoperability, we outlined additional steps that IDERHA has taken to further ensure findability, accessibility, interoperability, and reusability. These improvements, however, reflect the adherence to FAIR principles of IDERHA’s planned architecture and are based on a platform-level comparison. They will need to be confirmed through empirical validation once the IDERHA platform is fully implemented and operational across its federated sites. Despite these anticipated improvements, achieving them in practice will require careful attention to the necessity for well-defined metadata standards, as some data holders may encounter interoperability-related challenges or may be reluctant to share complete descriptions of their intellectual property for fear of losing control or facing competitive disadvantage. Furthermore, as the volume of data grows, managing a centralized metadata repository may become complex and resource intensive. Standards for characterizing a common data language that allows ML frameworks to describe and share models are still under development and not yet mature enough. Consequently, it is essential to develop semantic models and metadata within this domain to facilitate enhanced interoperability. When data is transferred between standards such as FHIR, CDISC SDTM, and OMOP-CDM, information loss has been reported across multiple real-world implementations. Differences between source and target models, gaps in vocabulary coverage, and missing or partial data in the source all contribute to records being dropped, approximated, or incompletely mapped during the conversion process, and no single standard fully meets all requirements for reliable data management and reuse. Using documented conversion workflows, quality assessment tools, and metadata frameworks to track transformation history is therefore important for ensuring data harmonization to support regulatory and decision-making.

In this work, we present the set of standards and recommendations we developed within IDERHA to support the secondary use of health data across multiple heterogeneous domains. We first identified and selected standards spanning clinical, imaging, socioeconomic, environmental, PREMs/PROMs, molecular and genetic data, as well as metadata, security, and privacy requirements. We then proposed a corresponding suite of standard-compliant data preparation and harmonization tools to support data providers during ingestion and transformation. To demonstrate their practical applicability, we implemented a lung cancer risk-assessment use case, showing how diverse data sources can be aligned with these standards and processed using the proposed tools. We further evaluated the feasibility of a subset of these tools through a pilot experiment on synthetic data, focusing on data quality and readiness assessment. Finally, we assessed the inherent adherence to FAIR principles of OMOP, clarifying its strengths and limitations, and showed how IDERHA’s infrastructure and metadata framework address these gaps to ensure stronger adherence to FAIR principles. Together, these contributions offer actionable guidance for projects aiming to enhance secondary health data use, improve interoperability, and align with the emerging requirements of the EHDS.

## Appendix

Multimedia Appendix 1 FAIRplusDSM assessment of OMOP data before IDERHA done in November 2025.

Multimedia Appendix 2 FAIRplusDSM assessment of IDERHA done in November 2025.

Multimedia Appendix 3 Overview of standard assesment in each domain.

Multimedia Appendix 4 DataHarmonizationResultsfromAchillesDQDAndCDMInspection.

## Abbreviations

AAI: Authentication and Authorization Infrastructure
ADaM: Analysis Data Model
BAM: Binary Alignment Map
BCF: Binary Call Format
CDISC: Clinical Data Interchange Standards Consortium
CDM: Common Data Model
CPT-4: Current Procedural Terminology, 4th Edition
CRAM: Compressed Reference-Aligned Map
DARWIN: Data Analysis and Real World Interrogation Network
DCAT: Data Catalog Vocabulary
DCAT-AP: DCAT Application Profile for European Data Portals
DICOM: Digital Imaging and Communications in Medicine
DQD: Data Quality Dashboard
DSM: dataset maturity
EHDEN: European Health Data & Evidence Network
EHDS: European Health Data Space
eID: electronic identification
eIDAS: Electronic Identification, Authentication, and Trust Services
EMA: European Medicines Agency
EMRN: European Medicines Regulatory Network
ESeC: European Socio-Economic Classification
ETL: extract, transform, and load
EU: European Union
EUCAIM: European Federation for Cancer Images
FAIR: findable, accessible, interoperable, and reusable
FHIR: Fast Healthcare Interoperability Resources
GA4GH: Global Alliance for Genomics and Health
GDI: Genomic Data Infrastructure
GIS: geographic information system
HL7: Health Level Seven
ICHOM: International Consortium for Health Outcomes Measurement
IDERHA: Integration of Heterogeneous Data and Evidence Towards Regulatory and Health Technology Assessment Acceptance
IHI: Innovative Health Initiative
INSPIRE: Infrastructure for Spatial Information in the European Community
LOINC: Logical Observation Identifiers Names and Codes
ML: machine learning
NIfTI: Neuroimaging Informatics Technology Initiative
OHDSI: Observational Health Data Sciences and Informatics
OMOP: Observational Medical Outcomes Partnership
ONNX: Open Neural Network Exchange
OPTIMA: Optimal Treatment for Patients With Solid Tumours in Europe Through Artificial Intelligence
PREM: patient-reported experience measure
PRO: Patient Reported Outcomes
PROM: patient-reported outcome measure
SAM: Sequence Alignment Map
SDOH: Social Determinants of Health
SDTM: Study Data Tabulation Model
SNOMED CT: Systematized Nomenclature of Medicine-Clinical Terms
TEHDAS: Towards the European Health Data Space (Joint Action)
UMLS: Unified Medical Language System
VA: Variant Annotation
VCF: Variant Call Format
VRS: Variation Representation Specification
W3C: World Wide Web Consortium

## Data Availability

The synthetic data used in this research are available upon request.
