# Supplementary material for: Bridging Gaps in Standards for the Secondary Use of Health Data: Exploratory Standard, Tool Assessment, and Feasibility Study
Source: JMIR Form Res. 2026 Sep 22;10:e88619. doi: 10.2196/88619 (PMC13643255; doi:10.2196/88619)
Supplement: Supplementary file 1 [file formative_v10i1e88619_app1.pdf]

FAIRplus

FAIR DataSet Maturity (FAIR-DSM) Assessment Tool

Level	Representation & Format	Content & Context	Hosting Environment Capabilities	Overall Level % Completion
Level 1	50%	0%	0%	21%
Level 2	50%	22%	0%	29%
Level 3	50%	44%	0%	36%
Level 4	17%	20%	0%	14%
Level 5	0%	0%	0%	0%

Based on this assessment, **11 indicators** still need to be satisfied for your Datasets to reach **Maturity Level 1**

- [x] [DSM-1-R2]Data intended for sharing and reuse have a purposely defined representation as Datasets
- [x] [DSM-1-R5]Dataset(s) available in Machine Readable Format
- [ ] [DSM-1-R0]Dataset Metadata is formally represented in the form of an Identifiable Dataset Descriptor
- [ ] [DSM-1-R3]A representation of the Dataset Descriptor conforming to a relevant General Purpose Metadata Schema is available
- [x] [DSM-1-R4]Dataset Descriptor is available in Machine Readable Format
- [ ] [DSM-1-R1]Contextual Metadata is represented at summary level and reported in the Dataset Descriptor
- [ ] [DSM-1-C0]Each Dataset purposed for sharing and re-use is assigned a unique identifier
- [ ] [DSM-1-C1]Dataset Descriptor(s) includes Descriptive Study/Project-Level summary information
- [ ] [DSM-1-C2]Dataset Descriptor(s) includes Identifying & Descriptive Dataset-Level metadata
- [ ] [DSM-1-C3]Dataset Descriptor(s) contains access information for the Dataset
- [ ] [DSM-1-H1]Metadata hosting environment stores and maintains an identifiable Dataset Descriptor for each identifiable Dataset
- [ ] [DSM-1-H2]The Dataset and its Descriptor are indexed and retrievable (in the same or separate hosting environments) via unique and persistent identifiers
- [ ] [DSM-1-H3]Retrieval of the Dataset and the Dataset Descriptor utilises a standardized communication protocol that is open, free and universally implementable
- [ ] [DSM-1-H4]Metadata hosting environment offers the capability to browse and search contents of the Dataset Descriptor

---

Based on this assessment, **15 indicators** still need to be satisfied for your Datasets to reach **Maturity Level 2**

- [x] [DSM-1-R2]Data intended for sharing and reuse have a purposely defined representation as Datasets
- [x] [DSM-2-R2]Dataset(s) are standardised to a locally defined Dataset Model suitable for data sharing and re-use
- [x] [DSM-1-R5]Dataset(s) available in Machine Readable Format
- [ ] [DSM-1-R0]Dataset Metadata is formally represented in the form of an Identifiable Dataset Descriptor
- [ ] [DSM-2-R3]Dataset Descriptor(s) adopt a Metadata Schema representation that describes the locally defined Dataset Model including its structural metadata (i.e. Field-level and Value-level metadata)
- [x] [DSM-1-R4]Dataset Descriptor is available in Machine Readable Format
- [ ] [DSM-2-R4]A formal documentation of the locally defined Domain Model is available in a Human Readable Format
- [ ] [DSM-2-R1]Contextual Metadata is formally represented and reported in the form of a locally defined Domain Model
- [x] [DSM-2-C2]Where applicable, data is structured in the Dataset according to the Tidy Data Principles
- [x] [DSM-2-C3]Dataset(s) include Reference Fields that enable joining related datasets
- [ ] [DSM-2-C4]Where applicable, Dataset Field Values are standardized against a locally defined Data Dictionary within and across related Datasets
- [ ] [DSM-2-C1]The locally defined Domain Model contains concepts that describes the overall project/study design, the relationships between the Datasets, the key entities reported within the Datasets and the relationships between them.
- [ ] [DSM-1-C2]Dataset Descriptor(s) includes Identifying & Descriptive Dataset-Level metadata
- [ ] [DSM-1-C3]Dataset Descriptor(s) contains access information for the Dataset
- [ ] [DSM-2-C5]Dataset Descriptor includes reference to related Datasets and if applicable the relevant joining Dataset Fields
- [ ] [DSM-2-C6]Dataset Descriptor includes Field-level Metadata as prescribed by a locally defined Dataset Model
- [ ] [DSM-2-C7]Dataset Descriptor includes Value-level Metadata or if applicable includes a reference to a locally defined Data Dictionary
- [ ] [DSM-2-H1]The Data hosting environment's Persistence Model is aligned with a locally defined Domain Model to enable interpretation of Datasets
- [ ] [DSM-1-H3]Retrieval of the Dataset and the Dataset Descriptor utilises a standardized communication protocol that is open, free and universally implementable
- [ ] [DSM-2-H2]Metadata hosting environment provides programmatic access and retrieval (API) for the Dataset Descriptor
- [ ] [DSM-2-H3]Data hosting environment offers the capability to browse and search related Datasets

---

Based on this assessment, **14 indicators** still need to be satisfied for your Datasets to reach **Maturity Level 3**

- [x] [DSM-1-R2]Data intended for sharing and reuse have a purposely defined representation as Datasets
- [x] [DSM-3-R2]Dataset(s) are standardised to relevant Domain or Community Standard Dataset Model(s) suitable for data sharing and re-use
- [x] [DSM-3-R5]Dataset(s) available in non-proprietary Machine Readable Format as prescribed by a standard Dataset Model
- [ ] [DSM-1-R0]Dataset Metadata is formally represented in the form of an Identifiable Dataset Descriptor
- [ ] [DSM-3-R3]Dataset Descriptor(s) use community-defined or domain-specific metadata standard
- [ ] [DSM-2-R4]A formal documentation of the locally defined Domain Model is available in a Human Readable Format
- [x] [DSM-3-R4]A formal documentation of the adopted Standard Dataset Model is available in a Machine Readable Format
- [ ] [DSM-3-R1]Contextual Metadata is formally represented and modeled according to a community or domain-specific standard
- [x] [DSM-2-C2]Where applicable, data is structured in the Dataset according to the Tidy Data Principles
- [x] [DSM-3-C2]Where applicable, Dataset(s) definition and content are reported in compliance with relevant community-defined Data Reporting Guidelines
- [x] [DSM-3-C3]Where applicable, Dataset Field Names use standard controlled terms as recommended by the adopted Standard
- [x] [DSM-3-C4]Where applicable, Dataset Field Values are standardised against domain-specific Controlled Terminologies and/or Ontology Terms
- [ ] [DSM-3-C1]Where applicable, study-level / experimental metadata is reported in compliance with relevant Minimum Information Reporting Guidelines
- [ ] [DSM-3-C7]Dataset Descriptor references a standard license under which the dataset can be re-used.
- [ ] [DSM-3-C6]Dataset Descriptor includes standard-compliant Field-level Metadata as prescribed by the adopted standard Dataset Model.
- [ ] [DSM-2-C7]Dataset Descriptor includes Value-level Metadata or if applicable includes a reference to a locally defined Data Dictionary
- [ ] [DSM-3-C5]Value Level Metadata includes Resolvable Identifiers for Controlled and/or Standard Terms reported in the Dataset
- [ ] [DSM-3-H1]The Data hosting environment's Persistence Model is aligned with a standard Dataset model or compliant with relevant Minimum Information Reporting Guidelines
- [ ] [DSM-1-H3]Retrieval of the Dataset and the Dataset Descriptor utilises a standardized communication protocol that is open, free and universally implementable
- [ ] [DSM-3-H2]For each dataset, the hosting environment maintains a globally unique, persistent and resolvable identifier for access and retrieval
- [ ] [DSM-3-H4]If applicable, Dataset hosting environment offers dataset-level authentication and authorisation capabilities
- [ ] [DSM-3-H3]Data Hosting environment utilises controlled terms and/or ontology terms to search within Dataset content.

Based on this assessment, **12 indicators** still need to be satisfied for your Datasets to reach **Maturity Level 4**

- [x] [DSM-1-R2]Data intended for sharing and reuse have a purposely defined representation as Datasets
- [ ] [DSM-4-R2]Dataset(s) are standardised to a defined Semantic Data Model and represented using Linked Data Represetations suitable for data sharing and re-use
- [ ] [DSM-4-R5]Datasets are available in a Machine Readable and Machine Interpretable format
- [ ] [DSM-4-R3]A Semantic Data Model (Metadata) used for data harmonisation across Datasets is formally defined and represented using Linked Data Representations
- [ ] [DSM-4-R4]A Semantic Data Model (Metadata) describing the data is represented in a Machine Readable and Machine Interptretable format
- [ ] [DSM-4-R1]Contextual Metadata is formally represented by a set of semantically defined Common Data Elements
- [ ] [DSM-4-C2]Dataset(s) content is harmonised against a designed-for-purpose Semantic Data Model
- [ ] [DSM-4-C3]Key Dataset Fields are mapped to Common Data Elements as defined by the Semantic Data Model
- [x] [DSM-4-C4]Values for key Domain Entities reported in the Dataset(s) are standardised and assigned unique Standard Identifiers
- [ ] [DSM-4-C1]A Semantic Data Model includes study design Data Elements and the relationships between them
- [ ] [DSM-4-C5]The Semantic Data Model includes a pre-defined set of Common Data Elements reported within the Datasets and the relationships between them
- [ ] [DSM-4-H1]Data Hosting environment stores data in a relevant linked data store (e.g., Triple Store or Graph Databaase)
- [ ] [DSM-4-H2]Data Hosting Environment provides semantic querying capability
- [ ] [DSM-4-H2]Data Hosting Environment provides semantic querying capability

Based on this assessment, **5 indicators** still need to be satisfied for your Datasets to reach **Maturity Level 5**

- [ ] [DSM-5-R2]Dataset(s) intended for sharing and re-use are granularly standardised and managed at the Data Element Level (e.g. ISO 11179 MDR standard)
- [ ] [DSM-5-R3]Common Data Elements and their value sets are defined and registered in a managed Metadata Registry
- [ ] [DSM-5-R1]Data content and context is defined and managed by Master Data Management
- [ ] [DSM-5-C3]Dataset Fields are linked and harmonized against enterprise managed Metadata Elements (e.g. MDR registered Data Elements)
- [ ] [DSM-5-C4]Dataset Field values are controlled and managed via enterprise managed Reference and Master Data

Print

FAIR Dataset Maturity model FAIR-DSM
